# The Role of Curcumin in Modulating Vascular Function and Structure during Menopause: A Systematic Review

**DOI:** 10.3390/biomedicines12102281

**Published:** 2024-10-08

**Authors:** Amanina Athirah Mad Azli, Norizam Salamt, Amilia Aminuddin, Nur Aishah Che Roos, Mohd Helmy Mokhtar, Jaya Kumar, Adila A. Hamid, Azizah Ugusman

**Affiliations:** 1 Department of Physiology, Faculty of Medicine, Universiti Kebangsaan Malaysia, Kuala Lumpur 56000, Malaysia; p132301@siswa.ukm.edu.my (A.A.M.A.); norizam_salamt@ukm.edu.my (N.S.); amilia@ppukm.ukm.edu.my (A.A.); helmy@ukm.edu.my (M.H.M.); jayakumar@ukm.edu.my (J.K.); 2 Cardiovascular and Pulmonary Research Group, Universiti Kebangsaan Malaysia, Bangi 43600, Malaysia; 3 Faculty of Medicine and Defence Health, National Defence University of Malaysia, Kuala Lumpur 57000, Malaysia; nuraishah@upnm.edu.my

**Keywords:** curcumin, endothelial dysfunction, menopause, ovariectomy, turmeric, vascular function

## Abstract

The risk of developing cardiovascular disease (CVD) escalates in women during menopause, which is associated with increased vascular endothelial dysfunction, arterial stiffness, and vascular remodeling. Meanwhile, curcumin has been demonstrated to enhance vascular function and structure in various studies. Therefore, this study systematically reviewed the recent literature regarding the potential role of curcumin in modulating vascular function and structure during menopause. The Ovid MEDLINE, PubMed, Scopus, and Web of Science electronic databases were searched to identify relevant articles. Clinical and preclinical studies involving menopausal women and postmenopausal animal models with outcomes related to vascular function or structure were included. After thorough screening, seven articles were selected for data extraction, comprising three animal studies and four clinical trials. The findings from this review suggested that curcumin has beneficial effects on vascular function and structure during menopause by addressing endothelial function, arterial compliance, hemodynamic parameters, and the formation of atherosclerotic lesions. Therefore, curcumin has the potential to be utilized as a supplement to enhance vascular health in menopausal women. However, larger-scale clinical trials employing gold-standard techniques to evaluate vascular health in menopausal women are necessary to validate the preliminary results obtained from small-scale randomized clinical trials involving curcumin supplementation (INPLASY, INPLASY202430043).

## 1. Introduction

Cardiovascular disease (CVD) is the leading cause of mortality worldwide. According to the World Heart Report in 2023 [1], CVD affects more than 500 million people globally. It was estimated that CVD was responsible for approximately 422.7 million cases and 17.9 million deaths in 2015, with the annual death toll expected to rise to 23.6 million by 2030 [2]. The increasing incidence of CVD risk factors, including hypertension, hypercholesterolemia, diabetes, overweight, and obesity, is the primary driver of CVD development.

Men are at a higher risk of developing CVD approximately 10 years earlier than women, while in women, the risk increases significantly after menopause [3]. CVD is the leading cause of mortality among women worldwide, accounting for approximately 35% of all female deaths globally [4]. This increased risk in menopausal women is believed to be due to the decline in estrogen levels [5]. The reduction in estrogen during menopause is associated with an elevated lipid profile, insulin resistance, and increased blood pressure, all of which contribute to vascular endothelial dysfunction and the subsequent development of atherosclerotic CVD [6].

Estrogen plays a crucial role in maintaining endothelial function, which is essential for cardiovascular health. It promotes vasodilation and suppresses oxidative stress and inflammation. However, the decrease in estrogen levels during menopause leads to endothelial dysfunction [5]. Endothelial dysfunction is characterized by impaired endothelium-dependent vasodilation, reduced nitric oxide (NO) bioavailability, and increased vasoconstriction, pro-inflammatory and pro-thrombotic states within the endothelium. These alterations contribute to the onset of atherosclerosis, disruption of blood flow, and an increased susceptibility to cardiovascular events [7].

Menopause is also associated with increased arterial stiffness and remodeling, marked by a reduction in the elasticity and compliance of arterial walls. As estrogen levels decline, arterial walls become less flexible, leading to changes in pulse wave velocity (PWV) and augmentation index (AIx) [8]. Increased arterial stiffness places additional strain on the heart and blood vessels, resulting in elevated systolic blood pressure (SBP), reduced coronary blood flow, and an increased workload on the cardiovascular system. These changes in arterial stiffness are implicated in the development and advancement of CVD, including hypertension and heart failure [8].

Several strategies can reduce the risk of CVD in menopausal women, including lifestyle changes and hormone replacement therapy (HRT). HRT is often chosen by menopausal women as it effectively relieves menopausal symptoms, prevents osteoporosis, and reduces the risks of CVD [9]. However, despite these significant benefits, HRT is associated with increased risks of certain cancers such as breast and endometrial cancer, as well as thrombosis and stroke [10,11]. Given the potential risks of HRT and the need for alternative approaches to prevent vascular dysfunction in menopausal women, various non-hormonal strategies, including the use of natural compounds such as curcumin, warrant exploration.

**Figure 1:**
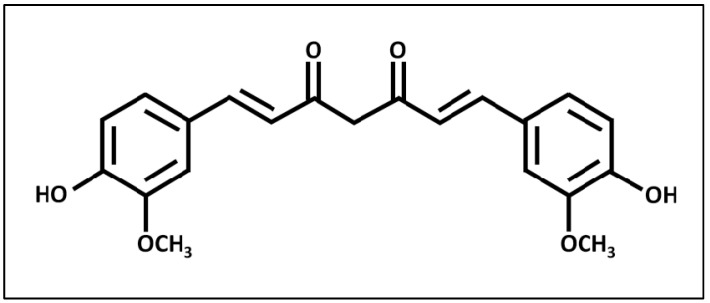
The chemical structure of curcumin.

Curcumin (1,7-bis-(4-hydroxy-3-methoxyphenyl)-1,6-heptadiene-3,5-dione) (Figure 1) is a hydrophobic polyphenol compound found naturally in the rhizome of *Curcuma longa*, commonly known as turmeric [12]. Turmeric consists of three curcuminoid compounds, namely curcumin, bisdemethoxycurcumin and demethoxycurcumin, with curcumin being the predominant component. Curcumin imparts the vibrant yellow color to turmeric [13]. Curcumin has been extensively researched for its potential therapeutic properties, including anti-inflammatory [13] and antioxidative effects [14]. It has also been shown to lower serum lipids and reduce the formation of atherosclerosis in low-density lipoprotein (LDL) receptor-deficient (LDLR^−/−^) mice [15]. Additionally, curcumin possesses antioxidant properties that mitigate adriamycin-induced cardiotoxicity and prevent cardiovascular events in individuals with diabetes [16]. Various animal models and clinical studies have firmly established the safety of curcumin as a compound, even at high doses of up to 12 g per day over a period of three months, with approval from the FDA [17,18,19].

Curcumin has the potential to reduce CVD risk in menopausal women by targeting vascular endothelial dysfunction [20]. However, no previous study has systematically reviewed the impact of curcumin supplementation on vascular function and structure during menopause. Therefore, this study was undertaken to systematically review the existing research on the role of curcumin in modulating vascular function and structure in the context of menopause. The findings from this review may provide valuable insights into the potential benefits of curcumin as an alternative treatment for reducing CVD risk in menopausal women.

## 2. Methods

### 2.1. Search Strategy

The literature search was performed across four electronic databases, including PubMed, Scopus, Ovid MEDLINE and Web of Science, utilizing the keywords curcumin AND (arterial stiffness OR aortic stiffness OR endothelial function OR vascular function OR blood pressure OR vascular structure) AND (menopause OR ovariectomy), covering the period from 1980 to 10 September 2024. This study complied with the Preferred Reporting Items for Systematic Reviews and Meta-Analyses (PRISMA) 2020 statement, ensuring a comprehensive and structured approach to the literature search, data extraction, and synthesis. The systematic review protocol was registered at the International Platform of Registered Systematic Review and Meta-analysis Protocols (INPLASY, INPLASY202430043) [21].

### 2.2. Study Inclusion and Exclusion Criteria

This review included original, full-length publications in English focusing on clinical and preclinical research investigating the role of curcumin on vascular function or structure in postmenopausal animal models or menopausal women, with consideration given to various routes of administration, doses, and intervention duration. Studies that involved mixed treatment regimens, where curcumin was combined with other agents or substances, were excluded. Additionally, studies involving non-postmenopausal animal models or clinical trials with non-menopausal women or mixed-gender cohorts were excluded. Furthermore, editorial letters, review articles, newsletters, case reports, conference proceedings, and book chapters were excluded from the review.

### 2.3. Study Selection and Data Extraction

The literature search and article screening followed the population, interventions, comparison, outcome, and study design (PICOS) framework, outlined as follows: population (P): postmenopausal animal models or menopausal women were included. Intervention (I): research involving curcumin as an intervention in the experimental group was included. Comparison (C): comparative groups either received no intervention or appropriate standard treatment. Outcome (O): outcome measures included changes in blood pressure, flow-mediated dilation, vasoreactivity, arterial stiffness and histology of the blood vessel. Study design (S): included preclinical (in vivo) and clinical (randomized controlled trial) studies.

The article screening process consisted of several steps. Initially, items that did not meet the selection criteria were eliminated solely based on their titles and types of publication (non-original article types such as abstracts, proceedings, reviews, study protocols, books, case reports, letters to the editor, meeting reports and commentaries were excluded). Next, studies unrelated to curcumin and vascular structure or function, as well as those not involving menopause models or subjects, were excluded after reviewing the abstracts. Subsequently, following a careful examination of the entire text, articles that failed to meet the inclusion requirements were excluded. Furthermore, using snowball search, the bibliographies of the included articles were manually checked for any additional studies that might have been missed during the database search. An independent review was conducted by three authors (A.A.M.A., N.S., and A.U.). Any disagreements were discussed until agreement was reached.

The study’s design, duration, and dose of curcumin supplementation were collected. Primary outcomes including vasoreactivity, arterial stiffness (PWV, AIx), SBP, diastolic blood pressure (DBP), mean arterial pressure (MAP), flow-mediated dilation (FMD), and blood vessel histology were extracted. Additional outcomes such as the levels of NO or other vasoactive substances and inflammatory markers were also extracted where available. If there was a lack of information or if the data were incomplete, an email was sent to the corresponding author to request any missing data as needed.

### 2.4. Risk of Bias Assessment

Two independent reviewers (A.A.M.A. and A.U.) conducted a comprehensive evaluation of potential bias, with any discrepancies resolved through consultation with a third reviewer (N.S.). The Cochrane risk of bias (RoB) tool [21] was used to evaluate the RoB in randomized clinical trials, while the RoB instrument developed by the Systematic Review Centre for Laboratory Animal Experimentation (SYRCLE) was used for assessing animal studies. Assessment criteria included the following: (1) selection bias, covering aspects such as random sequence generation, baseline characteristics, and allocation concealment; (2) detection bias, encompassing random housing, blinding, and random outcome assessment; (3) attrition bias, concerning incomplete outcome data; (4) reporting bias, addressing selective reporting; and (5) other bias [22]. Each domain was assessed to identify whether there was an unclear, low, moderate, or high risk of bias.

### 2.5. Statistical Analysis

The meta-analysis was performed using Review Manager (RevMan) 5.4 Software [23]. The mean difference (MD), along with its 95% confidence intervals (CI), was used to report the effect size of curcumin on brachial SBP and DBP. Heterogeneity between studies was assessed using two methods: (1) the Chi-squared test, with a *p*-value of less than 0.10 indicating a statistical significance; and (2) the Higgin’s I^2^ statistic [24]. An I^2^ value above 75% indicated high heterogeneity, 30–50% indicated moderate heterogeneity, and values below 25% indicated low heterogeneity. Due to the limited number of studies available for meta-analysis, a fixed-effect (FE) model was employed, and no subgroup analysis was performed. A funnel plot was not reported as less than ten studies were included in the meta-analysis. A sensitivity analysis was conducted using a random-effects (RE) model for the brachial blood pressure (BP) parameters to assess the robustness of the meta-analysis results.

## 3. Results

### 3.1. Studies Selected

**Figure 2:**
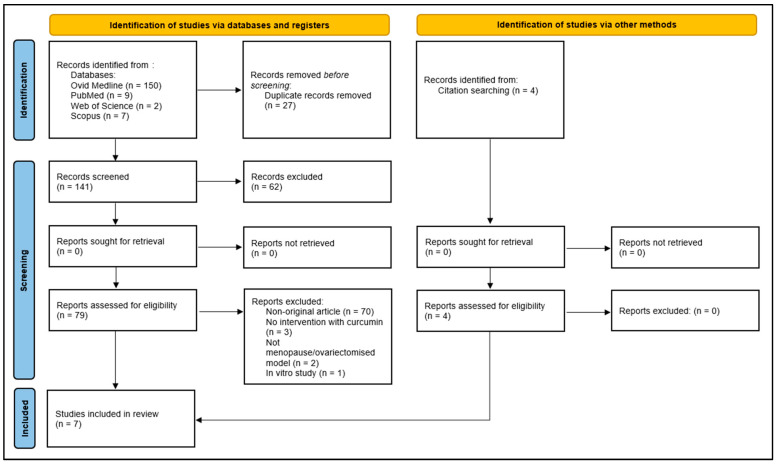
Preferred Reporting Items for Systematic Reviews and Meta-Analyses (PRISMA) 2020 flow diagram for systematic reviews.

Of the 168 records retrieved, 150 were sourced from Ovid MEDLINE, 9 from PubMed, 2 from Web of Science, and 7 from Scopus. A total of 27 duplicates were subsequently eliminated. Next, 70 non-original articles were excluded, leaving 71 original articles. After reviewing the titles and abstracts, 68 records were excluded for not meeting the selection criteria, such as not assessing vascular function or structure, using non-menopausal models, combining curcumin with other substances, or being in vitro studies. This resulted in three eligible records. Additionally, four records were included through snowball or manual searching, leading to a final set of seven full-length papers published between 2012 and 2022, all of which met the predefined selection criteria. The selection process is outlined in Figure 2.

### 3.2. Risk of Bias

**Figure 3:**
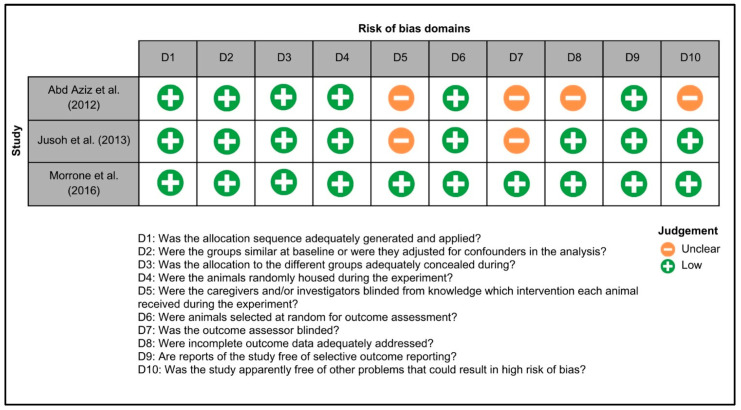
Systematic Review Center for Laboratory Animal Experimentation (SYRCLE) risk of bias summary: the reviewers’ assessments of the risk of bias for each item in every animal study included in the review, namely Abd. Aziz et al. 2012 [26], Jusoh et al. 2013 [27] and Morrone et al. 2016 [25].

**Figure 4:**
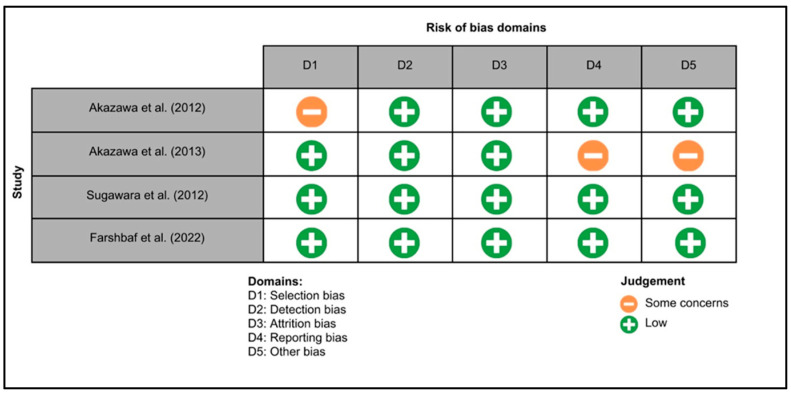
The Cochrane risk of bias tool for every randomized controlled trial included in the review, namely Akazawa et al. 2012 [28], Akazawa et al. 2013 [29], Sugawara et al. 2012 [30] and Farshbaf et al. 2022 [20].

Figure 3 summarizes the risk of bias in animal studies. One study exhibited a low risk of bias across all domains [25], while for two studies, the risks of bias related to blinding of caregivers, investigators and assessors, handling of incomplete data, and other potential sources of bias were unclear [26,27]. Figure 4 illustrates the risk of bias in clinical trials. Two studies exhibited a low risk of bias across all domains [20,28], while for two studies, the risk of selection, reporting and other biases were unclear [28,29].

### 3.3. Study Characteristic

The features of the selected studies are outlined in Table 1. The studies consisted of four clinical trials [20,28,29,30] and three in vivo animal studies [25,26,27]. The animal studies involved ovariectomized (OVX) Sprague Dawley rats fed with a high-cholesterol and heated palm oil diet [26], OVX Sprague Dawley rats fed with a high-cholesterol and heated soy oil diet [27], and OVX Wistar rats [25]. OVX rats were fed a high-cholesterol and heated oil diet to expedite the complications of menopause [26,27]. Two of the animal studies utilized curcumin C3 complex powder, a patented formulation containing a standardized mixture of curcumin, demethoxycurcumin, and bisdemethoxycurcumin, designed to enhance the bioavailability and absorption of curcumin [26,27]. Meanwhile, one study utilized non-formulated curcumin powder [25]. The dose of curcumin for the animal studies varied between 50 and 100 mg/kg/day, with a supplementation duration ranging from 1 to 4 months. None of the animal studies included a positive control group that utilized conventional drug or hormonal therapy.

Meanwhile, all clinical trials included healthy, non-smoking menopausal women [20,28,29,30]. None of the trials involved patients with diabetes mellitus, hypertension, coronary artery disease, or any significant peripheral arterial disease. Three of the clinical trials were conducted by the same group of researchers in Japan using different cohorts of menopausal women who were given curcumin pills dispersed with colloidal nanoparticles for better absorption [28,29,30]. One trial utilized curcumin capsules standardized to contain 475 mg of curcuminoid [20]. The dose used in clinical trials ranged between 150 and 1000 mg daily, while the duration of curcumin supplementation in all the clinical trials was eight weeks. None of the clinical trials included a positive control group that utilized conventional drug or hormonal therapy. However, three of the clinical trials included endurance exercise training [28,29,30], and one trial involved vitamin E supplementation for comparison [20].

### 3.4. Effect of Curcumin on Vascular Function in Menopause

Curcumin supplementation improved vascular endothelial function in postmenopausal women as evidenced by an increase in FMD [28]. Additionally, curcumin improved both peripheral and central arterial hemodynamics in menopausal women. For instance, curcumin supplementation reduced brachial [28,29] and carotid SBP [29] compared to pre-intervention levels. However, one study reported no change in brachial and aortic SBP following curcumin intervention [30]. Notably, none of the studies observed significant changes in brachial, carotid or aortic DBP with curcumin supplementation [28,29,30]. However, combining regular endurance exercise with daily curcumin ingestion successfully reduced aortic, carotid, and brachial SBP and DBP [29,30].

Regarding arterial compliance, subjects receiving curcumin supplementation demonstrated enhanced carotid arterial compliance, with improvements comparable to those observed with exercise training [29]. However, curcumin supplementation alone did not significantly alter the PWV, carotid AIx, radial AIx [30] or β-stiffness index [29]. While curcumin supplementation alone did not yield notable alterations in the PWV, carotid AIx, radial AIx or β-stiffness index, combining curcumin with regular exercise resulted in significant improvements in these measures in menopausal women [29,30]. Furthermore, curcumin supplementation significantly enhanced the biochemical markers of vascular function, as evidenced by reduced serum levels of high-sensitivity C-reactive protein (hs-CRP) in menopausal women [20] and decreased serum levels of interleukin-6 (IL-6) in OVX rats [25].

### 3.5. Meta-Analysis

**Figure 5:**
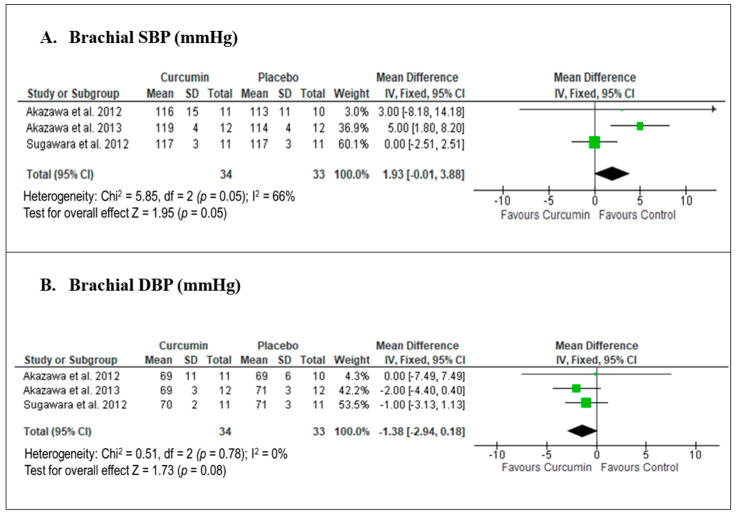
Meta-analysis of the effects of curcumin versus placebo on brachial blood pressure based on the findings of Akazawa et al. 2012 [28], Akazawa et al. 2013 [29] and Sugawara et al. 2012 [30]. The green square with horizontal line indicates individual study effect size (mean difference) together with its 95% confidence interval (CI), whilst the black diamond indicates the summary effect size together with its 95% CI.

Only three studies were included in our meta-analysis, all of which provided data for the pooled effects of curcumin on brachial SBP and DBP. A meta-analysis of these three studies [28,29,30] on the effect of curcumin versus placebo showed a marginally significant mean difference (MD) in brachial SBP, with an MD of 1.93 mmHg (95% CI −0.01, 3.88; *p* = 0.05; Figure 5A), and moderate heterogeneity was observed. In contrast, curcumin versus placebo demonstrated a non-significant reduction in brachial DBP, with an MD of −1.38 mmHg (95% CI −2.94, 0.18; *p* = 0.08; Figure 5B), and no heterogeneity was observed. A sensitivity analysis conducted using RE models showed comparable results to the primary meta-analyses, with MDs of 2.44 mmHg (95% CI −1.63, 6.50) for brachial SBP, and −1.38 mmHg (95% CI −2.94, 0.18) for brachial DBP.

## 4. Discussion

Findings from this systematic review suggested that curcumin has beneficial effects on vascular function and structure in both postmenopausal animals and clinical trials involving menopausal women. Improvement in endothelial function as measured by FMD was observed in menopausal women following eight weeks of curcumin supplementation. Curcumin supplementation alone demonstrated an improvement in FMD comparable to that achieved with exercise training [28]. FMD is a non-invasive method used to assess endothelial function by measuring the dilation response of an artery to increased blood flow. It is considered a key marker of vascular function, as the ability of blood vessels to dilate in response to shear stress depends on the release of NO from the endothelium [31]. As impaired FMD is an early indicator of endothelial dysfunction and atherosclerosis, curcumin’s positive effect on FMD suggests its potential to reduce cardiovascular risk, particularly by improving NO bioavailability and vascular function in postmenopausal women.

NO synthesized by endothelial nitric oxide synthase (eNOS) plays a crucial role in regulating blood pressure by promoting vasodilation. It also acts as an intrinsic anti-atherosclerotic agent by inhibiting monocyte adhesion, smooth muscle cell proliferation, and platelet aggregation [32]. A reduction in NO levels leads to endothelial dysfunction, which results in impaired blood pressure regulation and contributes to the development of atherosclerosis [31]. A previous study showed that 12 weeks of supplementation with 2 g of curcumin in healthy middle-aged and elderly men and menopausal women improved their conduit artery endothelial function and resistance. The improvements in endothelial function were attributed to a decrease in vascular oxidative stress and an increase in NO bioavailability [33]. Additionally, curcumin treatment restored endothelium-dependent vasorelaxation, increased eNOS protein levels, and decreased superoxide production in a homocysteine-induced endothelial dysfunction model of porcine coronary arteries [34]. Curcumin supplementation also lowered blood pressure and reduced vascular oxidative stress in 2-kidney-1-clip-induced hypertensive rats [35]. In terms of antioxidative action, curcumin directly scavenged free radicals [36] and decreased the expression of the reactive oxygen species (ROS)-producing enzyme NADPH oxidase while simultaneously enhancing the expression of the antioxidant enzyme superoxide dismutase [37].

Additionally, the anti-inflammatory effect of curcumin also contributes to its positive impact on the NO pathway and endothelial function. Curcumin supplementation has been shown to reduce hs-CRP levels in menopausal women [20] and IL-6 in OVX rats [25]. hs-CRP, an inflammatory marker, tends to be elevated in postmenopausal women [38]. CRP suppresses NO production by affecting eNOS activity [39]. This suppression occurs through the inhibition of guanosine triphosphate (GTP) cyclohydrolase 1 via the p38 kinase pathway, leading to reduced levels of the eNOS cofactor, tetrahydrobiopterin (BH_4_). The decrease in BH_4_ results in diminished NO levels, contributing to endothelial dysfunction [40].

Furthermore, curcumin downregulates TNF-α [41], which inhibits ROS generation and TNF-α-mediated signaling, thereby substantially reducing the risk of vascular dysfunction [42]. Curcumin also attenuated the activation of nuclear factor kappa B (NF-ĸB) induced by TNF-α, thereby decreasing the expression of pro-inflammatory cytokines and the generation of ROS. Additionally, it blocked the phosphorylation of signal transducer and activator of transcription (STAT)-3 and c-Jun N-terminal kinase (JNK) in human umbilical vein endothelial cells (HUVECs) [43]. Moreover, curcumin exhibited vascular anti-inflammatory effects by down-regulating toll-like receptors (TLR)-2 and -4 and reducing high mobility group box (HMGB)-1 protein levels in HUVECs [44]. Therefore, the overall findings suggest that the protective effects of curcumin on vascular endothelial function are mediated through improvements in NO bioavailability and reductions in vascular inflammation and oxidative stress.

Three clinical trials conducted by the same group of researchers in Japan investigated the effects of curcumin on central and peripheral arterial hemodynamic in different cohorts of menopausal women [28,29,30]. Central arterial hemodynamic parameters reflect the conditions in large arteries, such as the aorta and carotid arteries, which are closer to the heart and have a major influence on cardiac load and organ perfusion. In contrast, peripheral arterial hemodynamic parameters reflect the conditions in smaller, more distant arteries, such as the brachial and radial arteries, traditionally used in clinical practice to diagnose and manage hypertension and assess cardiovascular risk [45]. Although the three trials employed similar curcumin supplementation (150 mg curcumin pills dispersed with colloidal nanoparticles daily for eight weeks), the effects of curcumin on central and peripheral BP varied across the studies [28,29,30]. For peripheral (brachial) BP, Akazawa et al. (2012) [28] and Akazawa et al. (2013) [29] reported significant reductions in brachial SBP, while Sugawara et al. (2013) reported no significant change in brachial SBP [30]. Meanwhile, all three studies reported no significant changes in brachial DBP [28,29,30] or central (aortic) SBP and DBP [30].

The meta-analysis results suggest that curcumin has a marginally significant effect in reducing brachial SBP, with moderate heterogeneity observed, indicating some variability among the included studies. This variability may be attributed to differences in baseline participant characteristics, such as age, initial blood pressure levels, and overall cardiovascular health, which could influence individual responses to curcumin. However, curcumin did not demonstrate a statistically significant effect on brachial DBP, with no heterogeneity noted for DBP. Sensitivity analyses confirmed the consistency of these findings, suggesting that curcumin’s effect is more specific to SBP rather than DBP in peripheral arteries. SBP is more likely to respond to interventions that target the resistance in large arteries, such as the brachial artery, during heart contraction [46]. Curcumin enhances endothelial function by increasing NO production, promoting vasodilation and lowering SBP [33]. DBP, on the other hand, is more influenced by peripheral vascular resistance, governed by the smaller arterioles [46]. Curcumin may not exert a strong enough impact on peripheral resistance or the function of small arteries and arterioles to cause a significant reduction in DBP. Longer treatment durations and higher doses may be necessary to observe a significant impact on DBP.

Curcumin intervention also caused no significant change in the central (aortic) BP [30]. Central BP is considered more accurate than peripheral BP in predicting cardiovascular events and is more affected by arterial stiffness, which plays a significant role in aging and conditions such as atherosclerosis [45]. Eight weeks of curcumin supplementation did not induce significant changes in arterial stiffness in menopausal women, as measured by the PWV, AIx and β-stiffness index [29,30]. PWV is a widely used measure of arterial stiffness, indicating how quickly the pressure wave generated by the heartbeat travels through the arteries. Higher PWV values are associated with increased arterial stiffness, a strong predictor of cardiovascular events [31]. Menopause exacerbates the age-related increase in arterial stiffness, which is partly attributed to estrogen deficiency. Estrogen has a vasodilatory effect; thus, when estrogen levels decline, arteries may become stiffer [47]. Furthermore, the stiffness observed in large elastic arteries due to aging is influenced, in part, by structural alterations. These changes include increased deposition of collagen I, decreased elastin, and modifications of these proteins by advanced glycation end products (AGEs) [48].

Interestingly, animal studies have demonstrated that curcumin prevents cadmium-induced arterial stiffening in rats [49] and reduces arterial stiffness in hypertensive rats [50] and aged mice [51]. In aged mice, NO-mediated endothelial dysfunction and arterial stiffness, as assessed by aortic PWV, improved with just four weeks of curcumin supplementation. This improvement could be attributed to curcumin’s actions in decreasing oxidative stress and normalizing arterial collagen, elastin, and AGEs [51]. Despite these promising results from animal studies, no significant changes in arterial stiffness were observed in menopausal women, likely due to the short study duration of eight weeks, which may have been insufficient to induce any structural changes in the arteries. Further research is needed to determine whether long-term curcumin intervention can lead to improvements in arterial stiffness in menopausal women. Nevertheless, significant improvements in arterial stiffness and central BP were observed when curcumin was combined with regular exercise, suggesting that curcumin may improve arterial compliance more effectively in the presence of physical activity [29].

Curcumin supplementation mitigated the formation of atherosclerotic lesions in postmenopausal rats fed an HCD and heated palm oil, as evidenced by reduced disruption of endothelial cells and the internal elastic lamina, the absence of mononuclear cells in the intimal layer, and decreased migration of smooth muscle cells from the tunica media to the tunica intima [26]. The lack of estrogen in menopause induces the production of ROSs, which damage endothelial cells and contribute to the development of atherosclerosis [6,52,53]. Mechanistically, curcumin has demonstrated protective effects against oxidative stress and LDL modification by modulating lipid transport and preventing foam cell formation [54]. Curcumin reduces the expression of CD36, a cell surface receptor crucial for recognizing and internalizing oxidized LDL, thereby inhibiting the development of atherosclerosis [55]. Additionally, curcumin improves the LDL/high-density lipoprotein (HDL) cholesterol ratio, which is beneficial for preventing atherosclerosis [56]. Moreover, curcumin inhibits macrophage infiltration into atherosclerotic plaques by downregulating endothelial adhesion molecules and reducing coronary artery permeability [57]. Consequently, curcumin not only prevents the formation of atherosclerotic plaques but also stabilizes existing ones [56].

However, curcumin supplementation did not reduce atherosclerotic lesion formation in the aorta of OVX rats fed an HCD and heated soy oil [27]. The differential effects of curcumin on atherosclerotic lesion formation in postmenopausal rats fed an HCD with heated palm oil versus heated soy oil could be related to the composition of these oils. Palm oil contains high levels of saturated fats, particularly palmitic acid, which is highly atherogenic. The higher levels of saturated fats in palm oil contribute to more significant oxidative damage and elevated cholesterol levels, both of which are significant factors in atherosclerosis [58]. Curcumin can effectively reduce the oxidative stress and inflammation associated with this type of damage. In contrast, soy oil contains a higher proportion of polyunsaturated fats (PUFAs), such as linoleic acid. The PUFAs in soy oil are considered less atherogenic than the saturated fats in palm oil [59], which may explain why curcumin did not exert a noticeable protective effect, as the atherosclerotic lesion may not be as severe.

In animal studies, curcumin was administered at doses ranging from 50 to 100 mg/kg of body weight. In contrast, human studies used doses ranging from 150 to 1000 mg/day. The dose range of curcumin in animal models is significantly higher than that in human interventions, particularly on a per-kilogram basis. Standard curcumin extracts were used in the animal studies, which may necessitate higher doses to achieve plasma concentrations similar to those seen with nanocurcumin formulations in human studies. The bioavailability of orally administered curcumin is low due to poor absorption by the small intestine, extensive metabolism in the liver, and elimination through the gallbladder. Curcumin binds to enterocyte proteins, further decreasing its bioavailability as the structure of curcumin is modified [55]. Therefore, technological advancements addressing curcumin’s low bioavailability in the form of nano-formulations have been introduced.

Nanocurcumin formulations, such as Theracurmin^®^, are commonly used to enhance the bioavailability of curcumin, enabling therapeutic effects at lower doses [60]. Theracurmin^®^ disperses curcumin in colloidal submicron particles, significantly enhancing its bioavailability compared to conventional formulations [61]. For instance, a study demonstrated that Theracurmin^®^ provides a 27.3-fold higher concentration of curcumin over time compared to curcumin powder when both were administered at a 30 mg dose to seven subjects [62]. Additionally, increasing doses of Theracurmin^®^ from 150 mg to 210 mg resulted in higher plasma curcumin levels in healthy individuals, with no reported adverse effects [63]. Since nano-curcumin formulations have significantly higher bioavailability after oral administration compared to simple curcumin powder [64], formulated curcumin products like Theracurmin^®^ may have a more substantial impact on endothelial function than non-formulated curcumin [65].

## 5. Limitations and Future Recommendations

The variability in study designs, dosages, and curcumin formulations presents significant challenges when comparing results across studies. Different studies employed a wide range of curcumin doses, with animal models typically using higher doses (50–100 mg/kg/day) compared to human trials, where doses ranged from 150 to 1000 mg/day. Additionally, the formulations of curcumin varied considerably, impacting its bioavailability. While traditional curcumin powder was used in some animal studies, human trials often utilized nano-formulations like Theracurmin^®^ to enhance absorption and bioavailability. These differences in bioavailability may explain why higher doses were necessary in animal studies to achieve similar plasma concentrations observed in human trials. A suggested dose range for future human trials using nano-formulations such as Theracurmin^®^ could be between 180 and 360 mg/day [66].

Moreover, the short 8-week duration of the clinical trials may have contributed to the lack of significant efficacy of curcumin on arterial stiffness and some hemodynamic parameters. Future trials should explore longer-term interventions, lasting 3 to 6 months, as the current trials, limited to eight weeks, may be insufficient to detect significant changes. Additionally, the clinical trials were conducted with small sample sizes in each group, involving only healthy, non-smoking menopausal women without comorbidities, and lacked positive controls, such as hormonal therapy. Future studies should consider larger sample sizes and include more diverse populations, such as menopausal women with hypertension and dyslipidemia, to better understand curcumin’s efficacy in women with higher cardiovascular risk. Incorporating positive control groups using standard therapies, such as HRT, would allow for clearer comparisons of curcumin’s effectiveness.

Furthermore, more comprehensive biomarkers of vascular function should be measured in subjects, including biomarkers of inflammation and oxidative stress, vasoactive substances such as NO and endothelin-1, plasma curcumin concentration, and endothelial function assessed through indices such as the reactive hyperemia index and peripheral arterial tonometry. Collectively, these considerations underscore the need for a more comprehensive clinical trial investigation into the potential and safety of curcumin in treating menopausal women, with the goal of ameliorating the vascular dysfunction and remodeling often accelerated by menopause.

## 6. Conclusions

Curcumin supplementation improves vascular function and structure during menopause by addressing endothelial function, arterial compliance, hemodynamic parameters, and the formation of atherosclerotic lesions. Curcumin is suggested to enhance vascular health in menopause by leveraging its antioxidant and anti-inflammatory properties, collectively contributing to an increased bioavailability of NO. Therefore, curcumin has the potential to be utilized as a supplement to enhance vascular health in menopausal women. However, larger-scale clinical trials employing gold-standard techniques for evaluating vascular health in menopausal women are necessary to validate the preliminary results from small-scale randomized clinical trials.

## Figures and Tables

**Table 1:** Characteristics of selected studies on the effects of curcumin on vascular function and structure during menopause.

Experimental Model	Dose and Duration of Treatment	Findings	Outcome Measures	Reference
- In vivo study. A total of 30 ovariectomized (OVX) Sprague Dawley rats (200–250 g) fed a high-cholesterol diet (HCD) were equally divided into five groups (N = 6 per group): - Curcumin; - One-time heated palm oil + vehicle; - One-time heated palm oil + curcumin; - Five-times heated palm oil + vehicle; - Five-times heated palm oil + curcumin.	50 mg/kg curcumin C3 complex powder orally for 16 weeks	- Curcumin reduced atherosclerotic lesion formation in the aorta of OVX rats fed an HCD and heated palm oil. This was evidenced by less disruption of endothelial cells and the internal elastic lamina, the lack of mononuclear cells in the intimal layer, and reduced smooth muscle cell migration from tunica media to tunica intima. - Curcumin did not cause significant changes in the tunica intimal and medial thickness or the tunica intima-to-media ratio.	Curcumin reduces atherosclerotic lesion formation in the aorta of postmenopausal rats fed a high-cholesterol and heated palm oil diet.	[26]
- In vivo study. A total of 48 female Sprague Dawley rats (200–250 g) fed an HCD were equally divided into eight groups (N = 6 per group): - Sham-operated (Sham) + vehicle; - Sham + curcumin; - OVX + vehicle; - OVX + curcumin; - OVX + one-time heated soy oil + vehicle; - OVX + one-time heated soy oil + curcumin; - OVX + five-times heated soy oil + vehicle; - OVX + five-times heated soy oil + curcumin.	50 mg/kg curcumin C3 complex powder orally for 16 weeks	- Curcumin did not induce significant ultrastructural changes in the aorta of OVX rats fed an HCD and heated soy oil.	Curcumin does not reduce atherosclerotic lesion formation in the aorta of postmenopausal rats fed a high-cholesterol and heated soy oil diet.	[27]
- In vivo study. A total of 35 female Wistar rats (200–250 g) were divided into four groups: - Sham + vehicle (N = 8); - OVX + vehicle (N = 11); - OVX + curcumin 50 mg/kg (N = 8); - OVX + curcumin 100 mg/kg (N = 8).	50, 100 mg/kg curcumin powder intragastrically for 30 days	Compared with the untreated OVX group, OVX rats supplemented with curcumin had lower serum IL-6 levels (75.96 ± 0.56 vs. 78.80 ± 0.65 pg/mL, *p* < 0.05).	Curcumin reduces vascular inflammation in postmenopausal rats.	[25]
- Randomized controlled clinical trial. A total of 32 menopausal women (amenorrhea for at least 2 years) were randomly divided into 3 groups: - Control (N = 10, aged 64 ± 6 years); - Curcumin (N = 11, aged 60 ± 6 years, given curcumin); - Exercise (N = 11, aged 59 ± 5 years, underwent aerobic exercise training). All subjects maintained their regular diet. Subjects in control and curcumin groups maintained their regular physical activity, while the subjects in exercise group underwent aerobic exercise training more than 3 days per week for 8 weeks.	150 mg curcumin pills (dispersed with colloidal nanoparticles) daily, orally for 8 weeks	Compared with the control group, subjects supplemented with curcumin exhibited: - ↑ FMD; - ↓ brachial SBP (113 ± 11 mmHg vs. 116 ± 15 mmHg, *p* < 0.05). - no significant change in brachial DBP (69 ± 6 mmHg vs. 69 ± 11 mmHg, *p* > 0.05). The improvement in FMD and SBP with curcumin supplementation was comparable to exercise.	Curcumin improves vascular endothelial function in menopausal women in a manner similar to exercise.	[28]
- Randomized, double-blind parallel, placebo-controlled clinical trial. A total of 45 healthy, sedentary menopausal women (amenorrhea for at least 6 months) were randomly divided into 4 groups: - Placebo (N = 11, aged 59 ± 2 years, given dextrin and maltose); - Curcumin (N = 11, aged 61 ± 2 years, given curcumin); - Exercise + placebo (N = 11, aged 59 ± 2 years, given exercise training with dextrin and maltose); - Exercise + curcumin (N = 12, aged 60 ± 1 years, given exercise training with curcumin). All subjects maintained their regular diet. Subjects in the placebo and curcumin groups maintained their regular physical activity, while subjects in the exercise + placebo and exercise + curcumin groups underwent aerobic exercise training 3–6 days/week for 8 weeks.	150 mg curcumin pills (dispersed with colloidal nanoparticles) daily, orally for 8 weeks	Curcumin supplementation alone did not cause any significant change in the brachial and aortic SBP, DBP, PWV and AIx. Compared to their pre-intervention readings, subjects in the exercise + curcumin group had: - ↓ aortic SBP (107 ± 3 mmHg vs. 112 ± 2 mmHg, *p* < 0.05); - ↓ aortic DBP (69 ± 2 mmHg vs. 73 ± 2 mmHg, *p* < 0.05); - ↓ aortic AIx (17.4 ± 3.4% vs. 23.2 ± 1.3%, *p* < 0.05). Compared to the placebo and exercise + placebo groups, the exercise + curcumin group had ↓ radial Alx (83.3 ± 3.4% vs. 105.3 ± 6.4%, *p* < 0.05).	Combined curcumin ingestion and regular endurance exercise have more favorable effects on central arterial hemodynamic in menopausal women.	[30]
- Randomized, placebo-controlled clinical trial. A total of 51 healthy, sedentary menopausal women were randomly divided into 4 groups: - Placebo (N = 12, aged 58 ± 1 years, given dextrin and maltose); - Curcumin (N = 12, aged 60 ± 2 years, given curcumin); - Exercise + placebo (N = 13, aged 59 ± 2 years, given exercise training with dextrin and maltose); - Exercise + curcumin (N = 14, aged 60 ± 1 years, given exercise training with curcumin). All subjects maintained their regular diet. Subjects in the control and curcumin groups maintained their regular physical activity, while subjects in the exercise + placebo and exercise + curcumin groups underwent aerobic exercise training more than 3 days per week for 8 weeks.	150 mg curcumin pills (dispersed with colloidal nanoparticles) daily, orally for 8 weeks	Compared to their pre-intervention readings, subjects in the curcumin group had: - ↓ brachial SBP (119 ± 4 mmHg vs. 123 ± 5 mmHg, *p* < 0.05); - ↓ carotid SBP (108 ± 4 mmHg vs. 112 ± 5 mmHg, *p* < 0.05); - ↑ carotid arterial compliance (*p* < 0.05); - no significant change in brachial and carotid DBP, carotid IMT and β-stiffness index. Compared with their pre-intervention readings, subjects in the exercise + curcumin group had: - ↓ brachial SBP (113 ± 4 mmHg vs. 118 ± 4 mmHg, *p* < 0.05); - ↓ brachial DBP (67 ± 3 mmHg vs. 71 ± 3 mmHg, *p* < 0.05); - ↓ carotid SBP (102 ± 4 mmHg vs. 107 ± 5 mmHg, *p* < 0.05); - ↓ β-stiffness index (7.2 ± 0.4 U vs. 8.0 ± 0.5 U, *p* < 0.05); - ↑ carotid arterial compliance (*p* < 0.05); - no significant change in brachial and carotid DBP, carotid IMT and β-stiffness index. The combination of exercise training and curcumin ingestion led to a greater improvement in carotid arterial compliance compared to that achieved with either treatment alone.	Curcumin increases central arterial compliance in menopausal women. Combining curcumin with exercise is more effective at increasing arterial compliance than either curcumin ingestion or exercise alone.	[29]
- Triple-blind parallel randomized controlled clinical trial. A total of 81 healthy menopausal women (40 to 60 years old) were randomly assigned into 3 groups: - Curcumin (N = 26, aged 52.0 ± 2.5 years); - Vitamin E (N = 27, aged 52.5 ± 3.2 years); - Placebo (N = 28, aged 52.1 ± 2.7 years, given microcrystalline cellulose). All subjects maintained their regular diet and physical activity.	1000 mg curcumin capsules (standardized based on 95% turmeric root extract which contained 475 mg curcuminoid) daily, orally for 8 weeks	- Subjects supplemented with curcumin had ↓ hs-CRP (4.6 ± 2.6 vs. 5.1 ± 3. 4 mg/L, *p* = 0.025) compared with their pre-intervention readings.	Curcumin reduces inflammation in healthy menopausal women.	[20]

Abbreviations: ↑: higher; ↓: lower; Alx: augmentation index; BP: blood pressure; DBP: diastolic blood pressure; FMD: flow-mediated dilation; HCD: high cholesterol diet; hs-CRP: high-sensitivity C-reactive protein; IL-6: interleukin-6; IMT: intima-media thickness; OVX: ovariectomy; PWV: pulse wave velocity; SBP: systolic blood pressure; Sham: sham-operated.
